# Different time course recovery of muscle edema within the quadriceps femoris and functional performance after single- vs multi-joint exercises

**DOI:** 10.5114/biolsport.2023.119984

**Published:** 2022-11-18

**Authors:** Marco Aurélio Araújo Dourado, Denis C. L. Vieira, Daniel Boullosa, Martim Bottaro

**Affiliations:** 1 College of Physical Education, University of Brasilia – UnB, Brasilia, Brazil; 2 Centre d’Expertise de la Performance, Université Bourgogne Franche-Comté, UFR des Sciences du Sport, Dijon, France; 3 INSERM UMR1093-CAPS, Université Bourgogne Franche-Comté, UFR des Sciences du Sport, Dijon, France; 4 INISA, Federal University of Mato Grosso do Sul, Campo Grande, Brazil; 5 College of Healthcare Sciences, James Cook University, Townsville, Australia

**Keywords:** Strength training, Single joint exercise, Multi-joint exercise, Muscle damage, Torque impairments, Muscle recovery

## Abstract

This study aimed to verify the time course recovery of muscle edema within the quadriceps femoris and functional performance after lower-body single- and multi-joint exercises. For this within-participant unilateral and contralateral experimental design, fourteen untrained young males performed a unilateral knee extension exercise (KE), and a unilateral leg press (LP) exercise in a counterbalanced order. At pre-, post-, 24 h, 48 h, 72 h, and 96 h after exercise, the peak torque (PT), unilateral countermovement jump (uCMJ) performance, and rectus femoris (RF) and vastus lateralis (VL) muscle thicknesses were recorded in both legs. The PT decreased immediately after (p = 0.01) both exercises (KE and LP) and was fully recovered 24 h after KE (p = 0.38) and 48 h after LP (p = 0.68). Jump height and power, in the uCMJ, followed the same PT recovery pattern after both exercises. However, vertical stiffness (Kvert) was not affected at any time point after both protocols. The RF thickness increased after both exercises (p = 0.01) and was fully restored 48 h after KE (p = 0.86) and 96 h after LP (p = 1.00). The VL thickness increased after both exercises (p = 0.01) and was fully restored 24 h after LP (p = 1.00) and 48 h after KE (p = 1.00). The LP exercise, compared to KE, induced more prolonged impairment of functional performance and delayed recovery of RF muscle edema. However, the VL edema-induced muscle swelling recovery was delayed after the KE exercise. The different recovery kinetics between functional performance and muscle damage should be taken into consideration depending on the objectives of the next training sessions.

## INTRODUCTION

Resistance training (RT) is an important strategy for increasing muscle strength, muscle size, and neuromuscular functional capacity [1, 2]. However, one key variable that should be considered in the prescription of RT is exercise selection [3]. Among different classifications, resistance exercises can be divided into single- and multi-joint exercises. These exercises may induce different long-term RT adaptations (e.g., muscle strength and hypertrophy), and these outcomes may be mutually dependent [4]. However, when referring to lower-body exercises there is no consensus regarding the outcomes of the single- or multi-joint exercises. For instance, some studies have previously reported that the multi-joint leg press exercise may lead to greater improvements in the knee extensor strength than the single-joint knee extension exercise and suggested that these outcomes may be due to greater neural challenges in multi-joint exercises [5, 6]. In contrast, Stien et al. [7] reported that the knee strength improvements in single- and multi-joint exercises are task-dependent (i.e., improvements in knee extensor and leg press exercises strength will be greater in single- and multi-joint exercises, respectively).

Most studies comparing single- and multi-joint exercises have focused on chronic responses [4–6]. However, the acute specific responses of lower-body single- and multi-joint exercises to muscle damage and recovery remain under discussion [4]. It is well recognized that RT-induced muscle damage is accompanied by some acute deleterious effects such as neuromuscular function impairments (e.g., decrease in muscle force), delayed onset muscle soreness (DOMS), and edema-induced muscle swelling, and elevations in biomarkers such as creatine kinase [8–10].

Recently, Maeo et al. [10] reported that eccentric knee extension exercise (i.e. single-joint) induced a longer-lasting isometric peak torque impairment than the eccentric squat exercise (i.e. multi-joint). In addition, the authors observed greater muscle edema in the rectus femoris (RF) than the vastus muscles after eccentric knee extension exercise. In contrast, the vastus medialis showed greater muscle edema than the RF after eccentric squat exercise. In addition, Maeo et al. [10] also reported different recovery kinetics between isometric peak torque of the knee extensors and muscle edema in quadriceps muscles, which are indirect muscle damage markers, after single- and multi-joint lower-body eccentric exercises. However, the study by Maeo et al. [10] was performed with only eccentric actions, and it is well recognized that the magnitude of the induced muscle damage can be greater after eccentric than after concentric muscle actions [12, 13]. Thus, it is also important to investigate whether single- and multi-joint lower-body exercises performed during traditional RT using eccentric-concentric muscle actions would induce different knee extensor performance impairments and different magnitudes of muscle edema within the quadriceps (i.e., bi-articular RF vs. mono-articular VL).

Therefore, considering these aforementioned aspects, the purposes of this study were, first, to compare the time course recovery of indirect markers of muscle damage of the knee extensors; and second, to verify the different recovery kinetics in indirect markers of muscle damage of the knee extensors, after lower-body single- and multi-joint exercises. Based on previous evidence [2, 6, 11, 14], we hypothesized that leg press multi-joint exercise would induce greater muscle damage on the VL, and longer time course muscle performance recovery than the knee extension single-joint exercise.

## MATERIALS AND METHODS

### Study Design

**FIG. 1:**
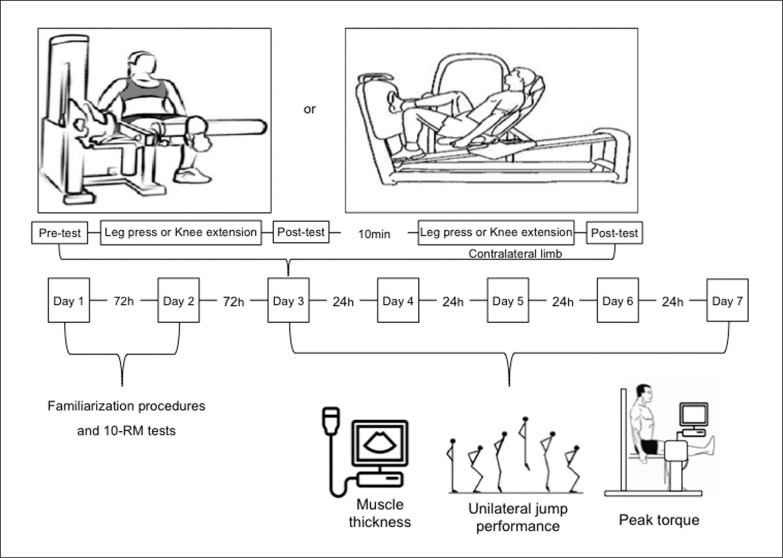
Study design.

The experimental procedures are shown in Figure 1. A within-participant unilateral and contralateral counterbalanced design was used to verify the magnitude and the time course recovery of muscle damage and functional performance after single- and multi-joint leg extension exercises. Participants visited the laboratory on eight occasions. On the first and second visits, the 10-maximum repetition (10RM) loads in unilateral leg extension and the unilateral leg press machine using the contralateral leg were determined. Moreover, the familiarization procedures with the unilateral exercises and neuromuscular evaluations (i.e., peak torque and jump performances) were completed. At the subsequent visit, participants performed the unilateral exercise protocols with an evaluation of unilateral maximal peak torque (PT), unilateral countermovement jump (uCMJ), and muscle thickness measured before, immediately after, and 24, 48, 72, and 96 h after the protocols. Participants were instructed to visit the laboratory at the same time of day on all occasions to avoid circadian influences. In addition, participants were not allowed to perform any vigorous physical activity or unaccustomed exercise during the experimental period.

### Participants

Fourteen healthy young men (age: 22.83 + 3.56 years; body mass 79.08 + 9.68 kg; height: 175.37 + 8.62 cm) completed all experimental sessions. To be included in this study, participants were required to have previous experience in RT with no exposure to resistance exercises for at least 6 months prior to study commencement, and to be free of cardiovascular, pulmonary, or metabolic disease. They were instructed to maintain their normal eating habits and to avoid alcohol, physical exercise, and interventions that could affect their recovery (i.e., massage, anti-inflammatory and analgesic drugs, and nutritional supplements). All procedures were approved by the University of Brasilia Committee on Human Research (CAAE No. 36351214.7.0000.0030) and were performed according to the Declaration of Helsinki. Informed consent was obtained from all participants.

### Ten-Repetition Maximum Tests

10 RM tests in the unilateral leg extension and leg press exercises were used to determine the loads to be used in the training session. Participants performed 10 RM tests adapted from the Brown et al. [15] recommendations. Briefly, they warmed up by performing two sets of 10 repetitions at 40% and 60% of their estimated 10RM, with a 60 s rest interval between the sets. After the warm-up, the 10RM load in each exercise was determined with no more than three attempts. The rest intervals were 5 min for each attempt, and 10 min between exercises. After 72 hours, a retest was performed to confirm 10RM loads and ensure data reliability.

### Strength Exercise Protocols

These within-subject training protocols were composed of 8 sets of 10 repetitions in the unilateral seated knee extension exercise (i.e., a single-joint exercise), and 8 sets of 10 repetitions in the unilateral leg press machine exercise (i.e., a multi-joint exercise). Each exercise was performed with a different leg in randomized order, with a 2 min rest interval between sets, and 10 min between exercises. The load of the 1^st^ to 3^rd^ set was 90% of the 10RM, and for the 4^th^ set onwards, the load was 70% of the 10RM. Participants were instructed to perform each repetition in approximately 4 s, with a concentric phase of 1–2 s and the eccentric phase of 2–3 s, and it was controlled verbally by the evaluator using a metronome.

### Maximal Isokinetic Knee Extension Torque

The time course of PT was used as an indirect measure of muscle damage to the knee extensors [8, 9]. The PT was recorded with an isokinetic dynamometer (Biodex Medical, Inc., Shirley, NY, USA) before, 10 min after, and 24, 48, 72, and 96 h after the exercise protocols. Participants were positioned and securely fastened in the dynamometer chair to minimize ancillary movements. The lever arm was fixed 3 cm above the lateral malleolus, and gravity correction was performed according to the manufacturer’s procedures. They performed 2 sets of 4 repetitions at 60°s^−1^ for each leg, with 2 min rest intervals between sets, and 10 min rest intervals between legs. The range of motion was set from 5º to 90º of knee flexion (0º = full extension). The same researcher carried out the procedures for all participants and provided verbal encouragement to achieve maximal performance in every attempt. Maximal PT was defined as the highest value of the torque curve recorded during a single unilateral knee extension repetition.

### Muscle Thickness Assessment

Muscle thickness was measured before, after 1 min, and 24, 48, 72, and 96 h after exercise protocols, via B-mode ultrasound (Philips-VMI, Ultra Vision Flip, model BF), which is a useful and valid tool to assess muscle edema [16]. For the measurements, participants were positioned supine on a table with the assessed knee supported from below to maintain 30° of flexion. Thereafter, they were asked to relax their limbs during the assessment. For baseline measurements, participants were evaluated in the supine position after 5 min of resting. A water-soluble transmission gel was applied on the 10 MHz ultrasound probe to provide acoustic contact without depressing the dermal surface. The ultrasound images were obtained from the middle points of the RF and vastus lateralis (VL) (i.e., 50% of the distance from the anterior superior iliac spine to the superior border of the patella) [11]. The measured sites were identified with a permanent marker at baseline to keep the same probe positioning after exercise, and for the subsequent exercise sessions. Ultrasound images were stored and further analysed using ImageJ software (National Institutes of Health, USA, version 1.49). Muscle thickness was defined as the distance between the border of the subcutaneous fascia and the deep aponeurosis [16]. All measurements were performed three times by the same investigator and the mean value was used for further analyses.

### Unilateral Countermovement Jump

The participants performed three maximum attempts, separated by > 15 s, of the unilateral countermovement jump (uCMJ) on a force plate (Advanced Mechanical Technology Inc., Watertown, MA, USA) that recorded vertical forces with a sampling rate of 500 Hz. The uCMJ was used because each exercise protocol was applied to a different limb for each participant. In addition, the uCMJ has been previously reported as a useful tool to assess acute neuromuscular performance changes after different exercises [17, 18]. Both exercise protocols and uCMJ order were performed with randomization of the limb used to avoid any interference of limb dominance and order on the results. Before each attempt, participants were instructed to stand still in the centre of the force plate with their hands placed on their hips. Subsequently, they were encouraged to jump “as high as possible”. The best attempt, based on jump height, was selected for further analysis. The uCMJs were performed before, after 10 min, and 24, 48, 72, and 96 h after the exercise protocols [9, 17]. The kinetic parameters of the best jumps were obtained with the corresponding software (AccuPower version 2.0, Massachusetts, USA) or calculated from the raw data in a custom-made Excel spreadsheet: jump height (h), determined from the difference between the maximum height of the centre of mass (apex) and the last contact of the toe on the ground during the take-off; peak power (PP) during the push-off phase (W · kg^−1^); and normalized vertical stiffness (Kvert) (N · m^−1^ · kg^−1^) (Kvert = Fmax · ΔY^−1^), where Fmax is peak vertical force minus body weight, and ΔY is the maximum vertical displacement of the centre of mass [19].

### Statistical Analyses

Data are presented as mean + standard deviation (SD) and 95% confidence interval. The Shapiro-Wilk test was used to check the normal distribution of variables. A two-way repeated measure ANOVA 2 × 6 (exercise × time) was used to compare muscle swelling, peak torque, and jump performance parameters between single- and multi-joint exercises. A Bonferroni *post hoc* correction was applied in the case of significant differences in the ANOVA. Cohen’s qualitative descriptors of standardized effect size were used to determine the magnitude of the exercise protocol changes and their recovery effects. The magnitude changes of the dependent variables before and after the exercise protocols were calculated from the formula: Post-test mean – Pre-test mean / Pre-test SD. In addition, the magnitude of the recovery parameters 24, 48, 72, and 96 h after the exercise protocols for each variable were calculated from: Post-24 mean (or 48, 72, 96) – Pre-test mean / Pre-test SD. The effect sizes (ES) < 0.4, 0.41–0.7, and > 0.7 represented small, moderate and large ES, respectively [20]. The significance level was set at p < 0.05. SPSS software (version 20.0; SPSS, Inc., Chicago, IL, USA) was used for all these analyses.

## RESULTS

### Peak Torque

The PT values are shown in Table 1. There was neither a significant effect for the factor exercise [F (1,13) = 0.76, p = 0.39] nor a significant exercise × time interaction [F (2.16, 28.11) = 0.77, p = 0.48] in the PT. However, there was a significant effect for the time factor [F (5,65) = 43.20, p = 0.01]. The Bonferroni post-hoc test revealed a significant decrease in PT immediately after both knee extension (p = 0.01) and leg press (p = 0.01) exercises. Moreover, PT was fully recovered 24 h after the knee extension exercise (p = 0.38), whereas it returned to baseline values 48 h after the leg press exercise (p = 0.68).

**TABLE 1:** Peak torque measures before and after both exercise protocols.

**Knee Extension**	PRE-	POST-	24 h	48 h	72 h	96 h

PT (Nm)	254.19 ± 29.32	206.44 ± 39.32*	242.23 ± 36.81	243.50 ± 35.00	250.21 ± 35.00	252.45 ± 26.96
95% IC	(237.3–271.0)	(183.7–229.1)	(220.9–263.4)	(223.2–263.7)	(230.0–270.4)	(236.9–268.0)
Effect Size	-	-1.63 (large)	-0.41 (moderate)	-0.36 (small)	-0.14 (small)	-0.14 (small)

**Leg Press**	PRE-	POST-	24 h	48 h	72 h	96 h

PT (Nm)	251.28 ± 40.75	197.15 ± 44.45*	236.04 ± 41.27*	238.44 ± 45.73	248.76 ± 44.67	252.69 ± 45.42
95% IC	(227.7–274.8)	(171.4–222.8)	(212.2–259.9)	(212.0–264.8)	(223.0–274.6)	(226.5–278.9)
Effect Size	-	-1.33 (large)	-0.37 (small)	-0.32 (small)	-0.06 (small)	-0.06 (small)

* Significant difference compared to the PRE moment with the same exercise protocol; PT = peak torque; CI = confidence interval; SD = standard deviation

### Muscle Thickness

The RF and VL muscle thicknesses are presented in Table 2. There was neither a significant effect for the exercise factor [F (1,13) = 2.10, p = 0.17] nor a significant exercise × time interaction [F (2.34, 30.45) = 1.35, p = 0.25] in the RF muscle thickness. However, there was a significant effect for the time factor [F (5,65) = 82.45, p = 0.01]. The Bonferroni post-hoc test showed that RF muscle thicknesses increased immediately after both knee extension and leg press exercises (p = 0.01) and returned to baseline values only after 96 h in the leg press exercise (p = 1.00) but after 48 h in the knee extension exercise (p = 0.86).

**TABLE 2:** Rectus femoris (RF) and vastus lateralis (VL) muscle thickness measures before and after both exercise protocols.

**Knee Extension**	PRE-	POST-	24 h	48 h	72 h	96 h

RF (mm)	25.21 ± 2.35	33.37 ± 2.10*	27.03 ± 2.94*	26.62 ± 3.27	26.07 ± 3.07	25.52 ± 2.59
95% IC	(23.87–26.58)	(32.15–34.58)	(25.33–28.73)	(24.73–28.51)	(24.30–27.84)	(24.03–27.01)
Effect Size	-	3.47 (large)	0.77 (large)	0.60 (moderate)	0.37 (small)	0.13 (small)
VL (mm)	24.22 ± 3.23	26.68 ± 2.94*	25.70 ± 3.27*	24.78 ± 3.18	25.06 ± 2.97	24.58 ± 3.20
95% IC	(22.36–26.09)	(24.97–28.38)	(23.81–27.59)	(22.95–26.62)	(23.35–26.78)	(22.74–26.43)
Effect Size	-	0.76 (large)	0.46 (moderate)	0.17 (small)	0.26 (small)	0.11 (small)

**Leg Press**	PRE-	POST-	24 h	48 h	72 h	96 h

RF (mm)	25.20 ± 2.53	32.94 ± 3.23*	27.37 ± 3.32*	27.23 ± 3.01	27.07 ± 1.95*	26.11 ± 3.00
95% IC	(23.74–26.66)	(31.08–34.80)	(25.46–29.29)	(25.49–28.97)	(25.94–28.19)	(24.38–27.84)
Effect Size	-	3.06 (large)	0.86 (large)	0.80 (large)	0.74 (large)	0.36 (small)
VL (mm)	25.01 ± 2.59	26.55 ± 2.28*	24.73 ± 2.86	24.66 ± 2.99	24.75 ± 2.51	24.89 ± 3.66
95% IC	(23.52–26.51)	(25.24–27.87)	(23.08–26.38)	(22.93–26.38)	(23.30–26.20)	(22.79–27.01)
Effect Size	-	0.59 (moderate)	-0.11 (small)	-0.14 (small)	-0.10 (small)	-0.05 (small)

* Significant difference compared to the PRE moment in the same exercise protocol; # significant difference compared to knee extension exercise. RF = rectus femoris; VL = vastus lateralis; CI = confidence interval; SD = standard deviation

For the VL muscle thickness, there was neither a significant effect for the exercise factor [F (1, 13) = 2.10, p = 0.85] nor a significant exercise × time interaction [F (4.07, 52.91) = 2.05, p = 0.09]. However, there was a significant effect for the time factor [F (5,65) = 82.45, p = 0.01]. The Bonferroni post-hoc test indicated that VL muscle thickness increased immediately after both knee extension and leg press exercises (p = 0.01). However, in the leg press exercise, the VL muscle thickness returned to baseline values after 24 h (p = 1.00), while it returned to baseline values after 48 h in the knee extension exercise (p = 1.00).

### Jump Performance

The jump height, PP, and Kvert during the uCMJ are presented in Table 3. There was neither a significant effect for the exercise factor [F (1,13) = 0.41, p = 0.54] nor a significant exercise × time interaction [F (2.76, 35.90) = 1.25, p = 0.31] in jump height. However, there was a significant effect for the time factor [F (5,65) = 17.65, p = 0.01]. The Bonferroni post-hoc test showed that jump height decreased immediately after both knee extension and leg press exercises. However, there was a different recovery profile between exercises. Jump height returned to baseline values after 48 and 24 h in the leg press (p = 0.15) and knee extension exercises (p = 0.32), respectively.

**TABLE 3:** Jump performance measures before and after both exercise protocols.

**Knee Extension**	PRE-	POST-	24 h	48 h	72 h	96 h

Jump (cm)	11.30 ± 3.11	8.69 ± 2.81*	10.14 ± 2.55	10.29 ± 2.92	10.52 ± 2.66	10.97 ± 2.35
95% IC	(9.51–13.10)	(7.07–10.31)	(8.67–11.61)	(8.60–11.98)	(8.99–12.06)	(9.62–12.33)
Effect Size	-	-0.84 (large)	-0.37 (small)	-0.32 (small)	-0.25 (small)	-0.11 (small)
Peak Power (W/kg)	32.32 ± 5.51	28.83 ± 5.06*	30.98 ± 4.94	30.27 ± 4.35	30.15 ± 3.44	30.94 ± 3.64
95% IC	(29.13–35.50)	(25.91–31.76)	(28.13–33.83)	(27.76–32.79)	(28.19–32.13)	(28.84–33.04)
Effect Size	-	-0.63 (moderate)	-0.24 (small)	-0.37 (small)	-0.39 (small)	-0.25 (small)
Kvert (N/m/kg)	104.34 ± 31.98	125.92 ± 43.81	108.09 ± 39.99	105.77 ± 28.68	96.14 ± 22.75	95.39 ± 22.12
95% IC	(85.87–122.80)	(100.63–151.21)	(85.00–131.18)	(89.21–122.33)	(83.00–109–28)	(82.62–108.16)
Effect Size	-	0.67 (moderate)	0.12 (small)	0.04 (small)	-0.26 (small)	-0.28 (small)

**Leg Press**	PRE-	POST-	24 h	48 h	72 h	96 h

Jump (cm)	11.41 ± 3.64	7.95 ± 2.44*	9.74 ± 3.16*	9.96 ± 2.74	10.58 ± 3.62	11.09 ± 3.00
95% IC	(9.32–10.50)	(6.54–9.37)	(7.92–11.57)	(8.37–11.55)	(8.49–12.67)	(9.35–12.82)
Effect Size	-	-0.95 (large)	-0.46 (moderate)	-0.40 (moderate)	-0.23 (small)	-0.09 (small)
Peak Power (W/kg)	31.21 ± 6.50	26.71 ± 5.47*	29.58 ± 6.68*	29.43 ± 6.07	30.52 ± 6.68	30.07 ± 5.35
95% IC	(27.46–34.97)	(23.55–29.87)	(25.73–33.42)	(25.93–32.94)	(26.66–34.38)	(26.98–33.16)
	-	-0.69 (moderate)	-0.25 (small)	-0.27 (small)	-0.11 (small)	-0.18 (small)
Effect Size	112.30 ± 36.78	129.39 ± 38.89	124.98 ± 77.14	133.08 ± 120.62	114.29 ± 37.68	99.86 ± 46.21
Kvert (N/m/kg)	(91.05–133.54)	(106.94–151.85)	(80.44–169.52)	(63.44–202.73)	(92.54–136.05)	(73.18–126.55)
Effect Size		0.46 (moderate)	0.34 (small)	0.56 (moderate)	0.05 (small)	-0.34 (small)

* Significant difference compared to the PRE moment in the same exercise protocol; Kvert = vertical stiffness; CI = confidence interval; SD = standard deviation

For PP, there was neither a significant effect for the exercise factor [F (1,41.51) = 1.39, p = 0.26] nor a significant exercise × time interaction [F (5, 4.71) = 1.98, p = 0.09]. However, there was a significant effect for the time factor [F (5, 47.61) = 11.80, p = 0.01]. The Bonferroni post-hoc analysis showed that in both exercises PP was immediately decreased, but with different recovery profiles. The PP returned to baseline values 24 and 48 h after the knee extension (p = 0.76) and leg press (p = 0.08) exercises, respectively.

For the Kvert, there was no significant effect for either the exercise factor [F (1,13) = 1.67, p = 0.22] or the time factor [F (5,65) = 3.03, p = 0.07] or a significant exercise × time interaction [F (1.35, 17.53) = 0.66, p = 0.47].

## DISCUSSION

The main finding of this study is that a fatiguing leg press exercise bout induced longer impairment of lower-body functional performance than the knee extension exercise. In addition, our results suggest that the leg press exercise may have induced greater RF muscle damage, as there was a longer recovery in RF edema-induced muscle swelling than the knee extension single-joint exercise. However, the VL muscle damage may be greater after knee extension exercise, as edema-induced muscle swelling recovery was longer in the knee extension exercise than in the leg press exercise. Moreover, the current findings also suggest that there were differences in recovery kinetics between indirect markers of muscle damage (i.e., functional performances and muscle-induced edema) after both exercises.

The leg press exercise induced a greater lower-body functional capacity impairment than the knee extension exercise, as confirmed by the delayed peak torque and jump height recovery. The greater impairment in the multi-joint exercise in the current study is contrary to the study by Soares et al. [8], who observed a greater peak torque impairment and longer time necessary for torque recovery in the elbow flexors after a biceps curl single-joint exercise than after the lat pull-down multi-joint exercise. In addition, Maeo et al. [10] reported a torque recovery of 48 h after an eccentric squat exercise, while the eccentric knee extension exercise showed a complete torque recovery in 72 h. These distinct responses could be due to differences in muscle structure and fibre type composition between upper and lower limb muscles [21]. Moreover, muscle action may also influence the extent of exercise-induced muscle damage [12, 13]. Therefore, the discrepancies between the current results and those of previous studies [9, 11] may be related to differences in muscles involved and type of muscle actions. Further studies are needed to test these hypotheses.

Several factors may affect the magnitude of exercise-induced muscle damage after fatiguing RT exercises, including the type of contraction, exercise load, volume, and velocity of contraction [8–10, 12, 13, 22, 23]. However, all these aforementioned factors were similar between protocols in the current study. In this regard, our results showed a longer period of torque impairment following the leg press exercise than the knee extension exercise, which may be related to the greater mechanical stress and higher neural activation imposed by the multi-joint exercise [6]. Nevertheless, further studies are needed to test these hypotheses.

An interesting result of the current study was a longer recovery in RF edema muscle swelling after the leg press exercise (i.e., 96 h) than knee extension exercise (48 h). Thus, these results may suggest that RF muscle damage is greater in lower limb multi-joint exercises because of the bi-articular nature of this muscle and the relationship between muscle lengthening and damage [24–26]. During the concentric phase of the leg press exercise, the distal portion of the RF may be gradually contracted to perform knee extension, but the proximal portion may be lengthened to induce hip extension, while the opposite happens during the eccentric phase. In contrast, in the knee extension exercise, the hip was always flexed, which in turn could limit the lengthening of the RF muscle [11]. Since muscle lengthening during an exercise is associated with greater force impairments and subsequent muscle damage [26], it may be expected that the leg press exercise could induce greater damage in RF muscle than the knee extension exercise, as confirmed in the current study.

Although the VL is a uni-articular muscle [27], which means that its activation cannot be influenced by different joint positions in both single- and multi-joint exercises [24, 25], this muscle showed a longer muscle edema recovery after the knee extension exercise than the leg press exercise. This finding suggests greater VL muscle damage in the single-joint exercise than after the multi-joint exercise. To the best of our knowledge, this is the first time this interesting finding has been reported. However, previous studies using electromyography have reported different hip joint positions’ influence on VL muscle activity [28, 29]. Indeed, Ema et al. [28] demonstrated higher excitation in the sitting position, followed by an inclined and supine position for the VL. Moreover, Lewek et al. [30] previously reported a greater VL muscle activity in the knee extension exercise than in the lunge exercise. Therefore, it may suggest greater action and mechanical stress of the VL muscle when the hip is flexed during knee extension movements, with further studies needed to confirm our findings.

In the current study, the jump and torque performances in both exercises exhibited the same recovery pattern. Nonetheless, muscle edema recovery was delayed after both protocols. Similar to our study, Maeo et al. [11]reported a full torque recovery 24–48 hours after eccentric knee extension, squat, and downhill walking exercises. Meanwhile, the time course recovery of vastus muscles and RF was longer than 72 hours after these exercises. Thus, the results of the previous [11] and the current study may indicate that muscular performance can be fully recovered in a shorter time despite the pro-inflammatory responses induced by muscle swelling [31]. In this regard, other factors may be influencing muscular performance recovery after fatiguing exercise. For instance, it has been suggested that the perception of fatigue, muscle soreness, and stiffness may influence both muscular performance and subsequent recovery after eccentric exercises [31]. While future studies should be conducted to appropriately test this hypothesis, independently of the associated mechanisms, the earlier recovery of muscular performance is relevant because muscle strength and power can be associated with functional performance [32].

Finally, the post-measures for Kvert did not show significant differences from pre-exercise measures in both exercises. However, considering the ES, Kvert and jump performance revealed a different time course with preservation of stiffness despite the impairments in jumping performance. It has been proposed that a high stiffness is favourable to improve performance in movements involving the stretch-shortening cycle [33]. Moreover, an association was previously reported [19] between Kvert and the rate of force development (RDF) during the CMJ, thus confirming the important role of Kvert in vertical jumping. Considering the current and the aforementioned studies [19, 33], the existence of a compensatory neuromuscular mechanism related to this Kvert preservation may be suggested. However, this should be interpreted with caution because these above-mentioned studies [19, 33] have a cross-sectional design. Similar to the current study, a previous study reported an increase in passive muscle stiffness after a fatiguing exercise, which may provide additional information on the recovery status of subjects [34]. Therefore, further studies should be conducted to verify whether the acute adaptations in stiffness from different exercises are associated with a compensatory mechanism linked to a recovery status.

Our study has some limitations. First, no measures of delayed onset muscle soreness were included; these might have helped to better explain the dissociated responses between functional performances and edema-induced muscle swelling. In addition, no electromyographic measures were recorded, though it would provide valuable information on the active muscles during both fatiguing and evaluation protocols.

## CONCLUSIONS

The current findings indicate greater mechanical stress in the leg press exercise than in the knee extension exercise, as demonstrated by longer impairments in torque, power, and jump performance. The current results may explain the greater strength improvements after resistance training with multi-joint exercises [6]. In addition, considering the bi-articular nature of the RF [24, 25] and the relationship between muscle lengthening and muscle damage [26], it may be suggested that the RF is more damaged after the leg press exercise than the knee extension exercise. In contrast, it seems that the VL is more stressed when the hip joint is flexed, as occurred in the current and previous studies [28, 29] that investigated the hip angle position during knee extension exercise. Finally, the faster recovery of functional performance despite the presence of muscle damage should be taken into consideration depending on the objectives of the next training sessions (e.g. hypertrophy vs. power).
